# Supplementary material for: JcvPCA and JsvCRP: A set of metrics to evaluate changes in joint coordination strategies
Source: PLoS One. 2025 Aug 5;20(8):e0325792. doi: 10.1371/journal.pone.0325792 (PMC12324137; doi:10.1371/journal.pone.0325792)
Supplement: S1 File — This file contains the details of all mathematical notations used in this article. (PDF) [file pone.0325792.s001.pdf]

# Mathematical Notations

This Appendix summarizes all the mathematical notations used in this work to describe the two metrics.

- The variable $t$ represents the time elapsed during the motion. The motion begins at time $t_0$ and concludes at $t_{mvmt}$. In instances of normalized temporal movements, $t_{mvmt}$ corresponds to 100% of the total duration
- $n \in \mathbb{N}$ is the number of considered number of degrees of freedom (i.e. joints).
- $p \in \mathbb{N}$ with $p \leq n$ is the dimensionality of the task. There are at least as many joints as the dimensionality of the task in order to be able to perform it. There might be more joints than required for the task, creating redundancy and leading to different possible coordination strategies.
- A and B designate the variables that relate to dataset A or dataset B. Each dataset is composed of sub-sets of data movements of $n$ joint trajectories. The aim is to test whether A and B demonstrate similar or different control strategies.
- $i$, $j$ are the names of the 2 different joints from the same dataset with $i \leq n$ and $j \leq n$
- $\theta \in \mathbb{R}^{\mathrm{n}}$ are the joint trajectories, where $n$ is the number of joints
- $\dot{\theta} \in \mathbb{R}^{\mathrm{n}}$ the joints' angular velocity
- $\phi \in \mathbb{R}^{\mathrm{n}}$ the joints' phase angle
- $k$ and $l \in \mathbb{N}$ are the number of movements contained in respectively dataset A and dataset B
- $m \in \mathbb{N}$ is the number of considered PC. $m$ can be chosen between $p \leq m \leq n$. Here we suggest selecting $m$ as $m = p + 1$. Thus, the last PC will provide grouped information about the null space, while the first PCs will contain information related to task execution and the amount of explained variance with m PCs should be sufficient to explain at least 90% of the dataset's variance.
- $CRP_{i,j} \in \mathbb{R}^{\frac{\mathrm{n}!}{2!(\mathrm{n}-2)!}}$ the CRP computed between joints i and j
