# Supplementary figures and images for: JcvPCA and JsvCRP: A set of metrics to evaluate changes in joint coordination strategies
Source: PLoS One. 2025 Aug 5;20(8):e0325792. doi: 10.1371/journal.pone.0325792 (PMC12324137; doi:10.1371/journal.pone.0325792)

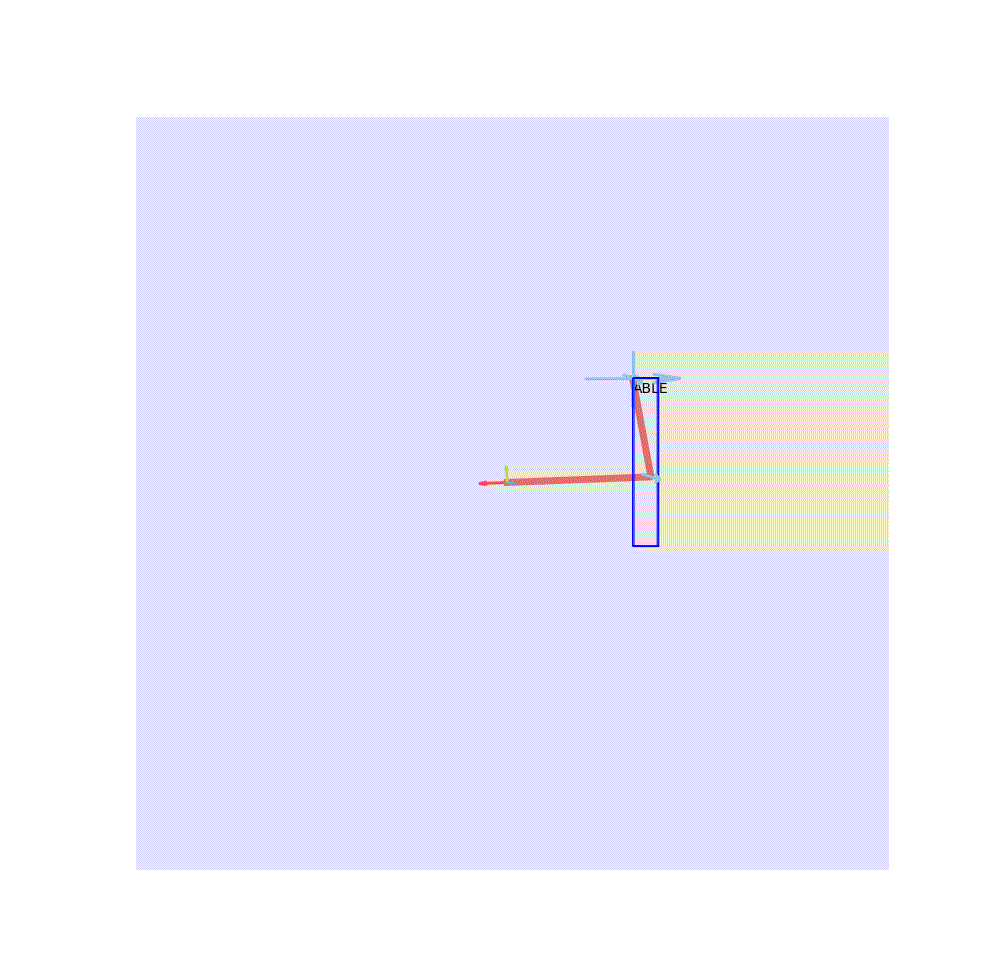

Supplement: S3 Video — This is a video of an animation of the 4 different coordination strategies (Physiological, Shoulder Only, Overuse of the elbow, Temporal Desynchronization) that have been experimentally recorded, replayed with a stick figure. (ZIP) [file pone.0325792.s003.zip › overuse_elbow.gif]

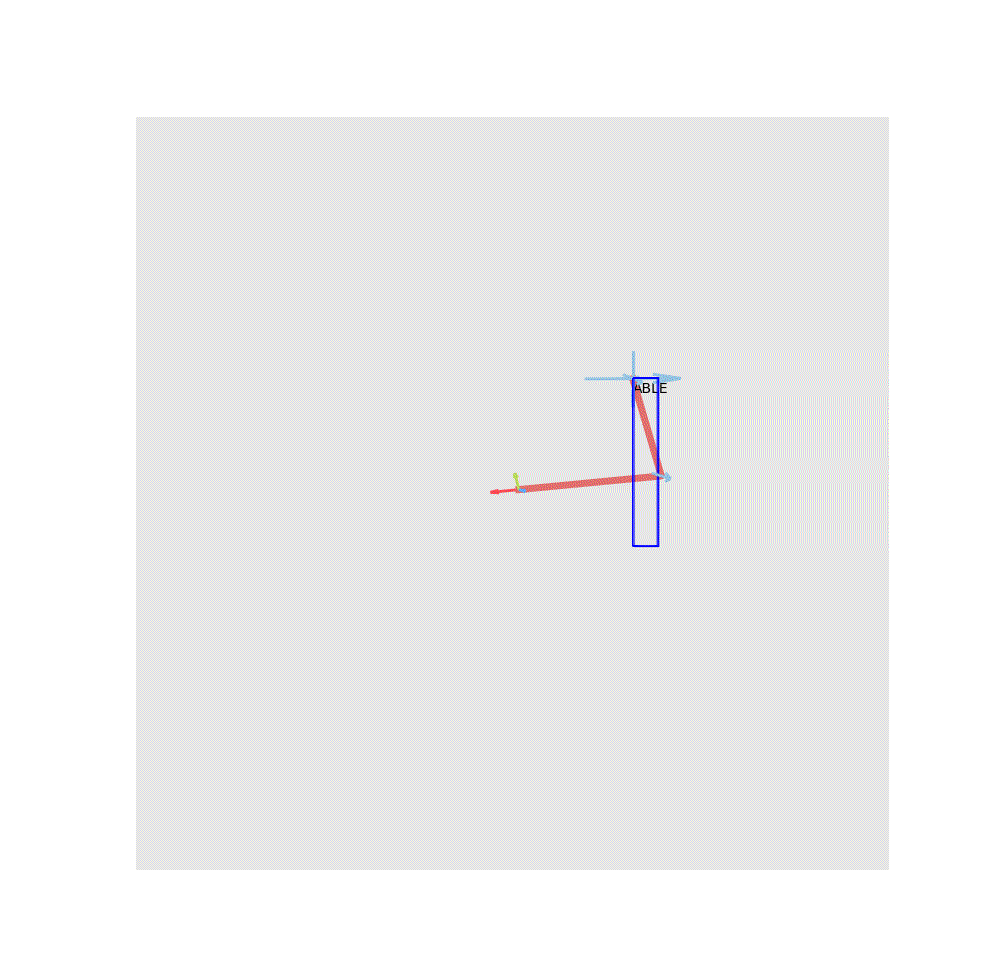

Supplement: S3 Video — This is a video of an animation of the 4 different coordination strategies (Physiological, Shoulder Only, Overuse of the elbow, Temporal Desynchronization) that have been experimentally recorded, replayed with a stick figure. (ZIP) [file pone.0325792.s003.zip › physiological.gif]

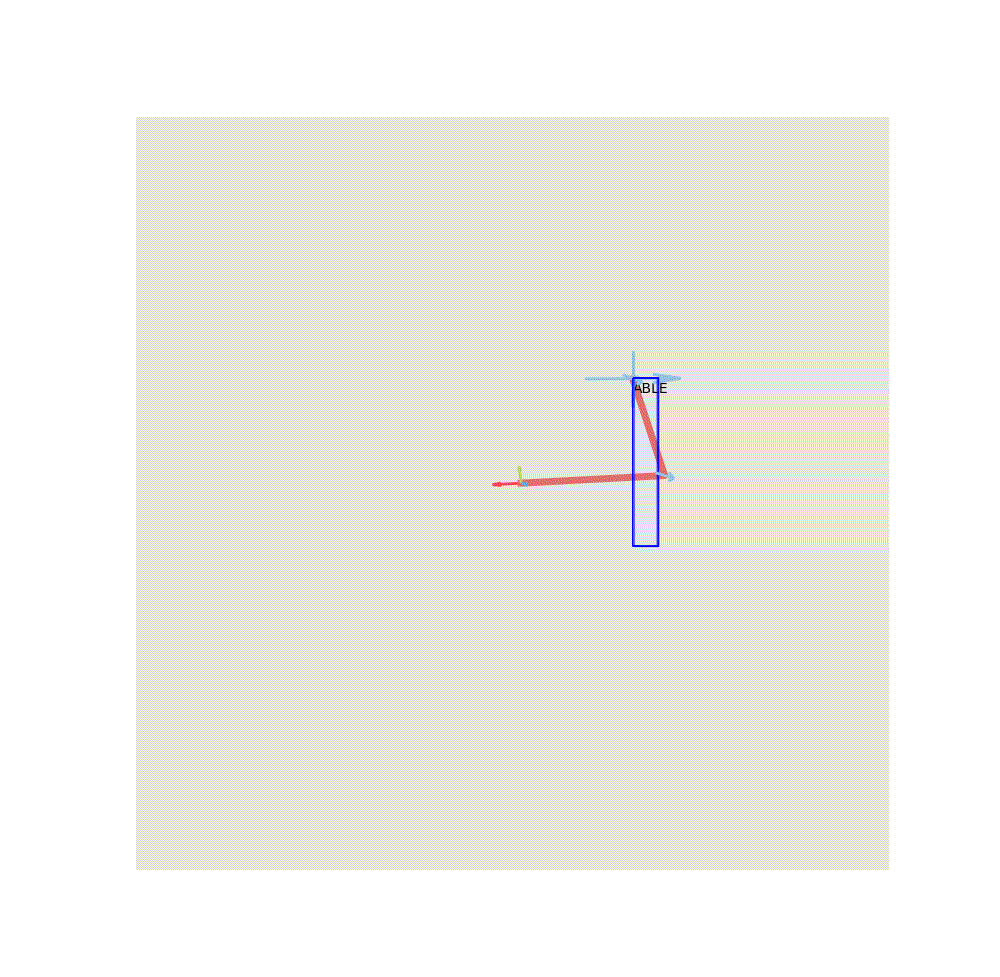

Supplement: S3 Video — This is a video of an animation of the 4 different coordination strategies (Physiological, Shoulder Only, Overuse of the elbow, Temporal Desynchronization) that have been experimentally recorded, replayed with a stick figure. (ZIP) [file pone.0325792.s003.zip › shoulder_only.gif]

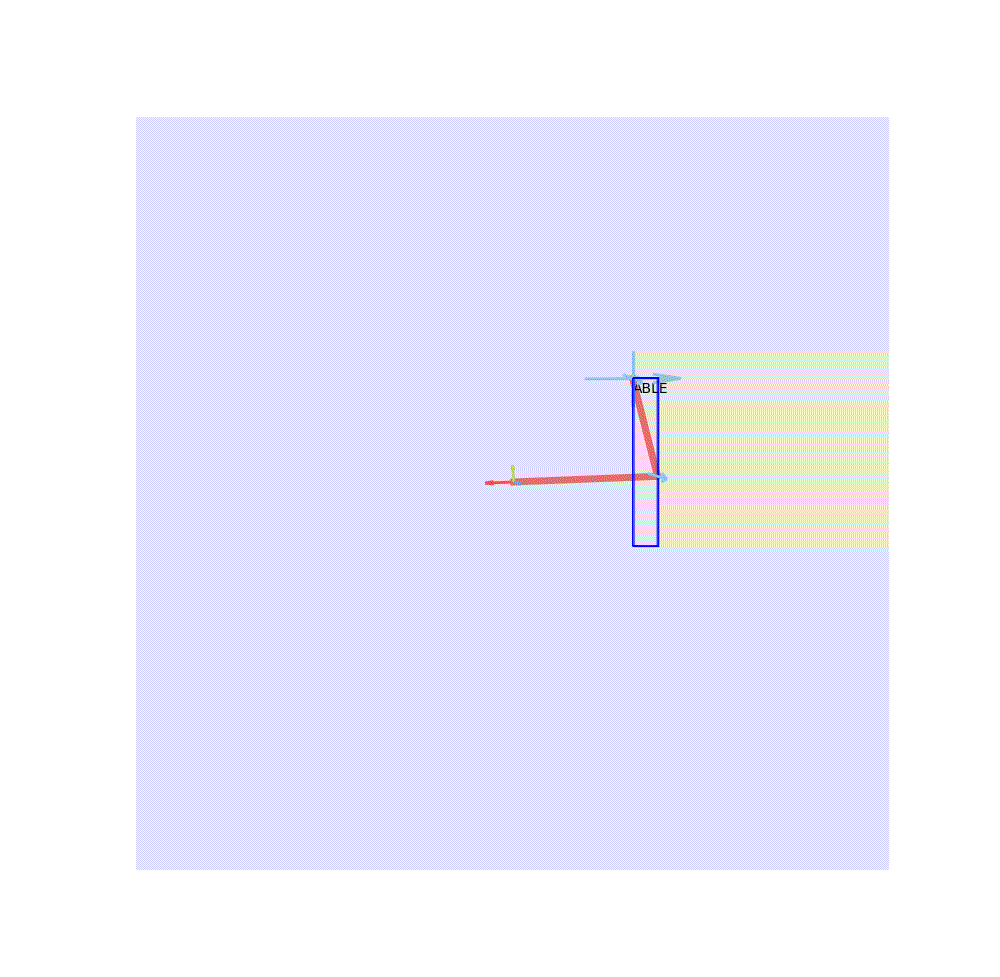

Supplement: S3 Video — This is a video of an animation of the 4 different coordination strategies (Physiological, Shoulder Only, Overuse of the elbow, Temporal Desynchronization) that have been experimentally recorded, replayed with a stick figure. (ZIP) [file pone.0325792.s003.zip › temporal_desynchronization.gif]
