# Supplementary material for: JcvPCA and JsvCRP: A set of metrics to evaluate changes in joint coordination strategies
Source: PLoS One. 2025 Aug 5;20(8):e0325792. doi: 10.1371/journal.pone.0325792 (PMC12324137; doi:10.1371/journal.pone.0325792)
Supplement: Attachment — Submitted filename: RebutalLetter.pdf [file pone.0325792.s005.pdf]

Dear Dr. Roberto Di Marco,

Thank you for your response and for considering our manuscript for publication. We appreciate the opportunity to revise and resubmit our work to address the comments raised by the reviewers.

We have carefully reviewed the feedback provided by the academic editor and the reviewers. We acknowledge the importance of addressing the reporting advantages and limitations of the method proposed in our manuscript, particularly in comparison to other existing methods. In our revised manuscript, we ensure to provide a comprehensive discussion on these aspects, highlighting the strengths and weaknesses of our approach in comparison to alternative methods.

Here are listed, point by point, the review and our response to the review :

JOURNAL REQUIREMENT

Report advantages and limitations of using this method, rather than others.

The advantages and limitations of this method have been enhanced in paragraph 1.1, 5.3, and especially in paragraphs of section 6.1.

Please state what role the funders took in the study.

The funders had no role in study design, data collection and analysis, decision to publish, or preparation of the manuscript.

Please expand the acronym “ANR” (as indicated in your financial disclosure) so that it states the name of your funders in full.

ANR stands for Agence National de la Recherche and is the French National Agency that funds and promote research in France.

FIGURE 1 ET 2 : Please clarify whether this [conference proceeding or publication] was peer-reviewed and formally published. If this work was previously peer-reviewed and published, in the cover letter please provide the reason that this work does not constitute dual publication and should be included in the current manuscript.

Figure 1 and 2 where peer-reviewed by reviewer for the International Conference on Robotics and Automation (ICRA) 2024. This proceeding will be pubished after the conference that took place in Japan from the 13th to the 17th of May. Thos figures are used to explain briefly how the metrics are computed, since we use them in the paper but they are not published yet. The proceeding of the conference then developps the study of the impact of exoskeletons on inter-joint coordination, using those metrics. The paper that we submitted here really goes into details on how to compute de metrics, developping all the equations, and then testing the metrics, both on simulated and experimental datasets. This paper also provides hints on how to interpret the metrics’ results.
The proceedings of the conference have already been published and will be added to the attached file with the manuscript.

Please include your full ethics statement in the ‘Methods’ section of your manuscript file. In your statement, please include the full name of the IRB or ethics committee who approved or waived your study, as well as whether or not you obtained informed written or verbal consent. If consent was waived for your study, please include this information in your statement as well.

In the method section, the full name of the ethic comitee as well as the protocol approval number was added

Include captions for your Supporting Information files at the end of your manuscript, and update any in-text citations to match accordingly.
The caption of S3 have been added and the in-text citations of the 3 Supporting files have been corrected.

REVIEWER 1
Please add numerical values, a description of the results and conclusions to the abstract, the abstract does not have the correct form - it needs to be reworded
Abstract have been reworded. However no numerical values have been added since here a method is presented we do not have numerical result to present in the abstract.

Is examining such a small number of people objective? please justify your answer?
This article aims at illustrating a possible usecases of this metric. The experiment including 1 subject is here to validate that the results obtained with those metrics are still valid when using an experimental dataset, recorded on human, thus including more variability than our simulated dataset. Moreover, in this experimental dataset we specifically asked the participant to perform different inter-joint coordination strategies to verify if the metrics were able to enhance the differences between the different coordinations. This first exemple seems to be concluant and let us think that this metric could now be extended to larger goups.
In the article, we've highlited that this experimental dataset is here to serve as an illustration to how the metrics could be used in physiological conditions. This have been clarified at the beginning or section 5.

3. Can the presented data be compared numerically? please justify your answer
All the presented data can be compared numerically since the reference dataset and the experimental conditions are the same between all conditions. This point has been clarified in the introduction of section 5.2.

4. The results do not properly present the numerical values obtained during the study - the presentation of the areas under the graphs does not properly reflect the differences? Were average values used for comparison? why average if the sample is so small?
In order to obtained a more robust CRP, all movements (5 times each 3 targets) are averaged together. This is done so a small variation in one of the movements do not influence too much the overall result. In the end, one CRP curve is the average of all the movements so we get the global trend of the movement.
In addition, the section 5.2.1 have been added to explain how natural variability of the metric can be computed and give numerical values of this variability. All values that is above this threshold can be considered as a difference of coordination strategy.

5. Why were these particular strategies chosen to execute the moves?
The reasons that we've choosen those particular strategies are described in section 5.1.2. The idea is to have different, easy to perform, strategies that can test the ability of the metrics to evaluate both a change in temporal or in spatial inter-joint coordination. We therefore have the baseline condition which is the physiological one, and then 1 that tests the temporal aspect of inter-joint coordination, which is the desynchronization strategy. 1 that tests the spatial aspect of inter-joint coordination which is the overuse of the elbow and one final one that tests both aspects which in the use of the shoulder only.

6. what does it mean that the curves are different from each other? Do we have any numerical values confirming that there are differences? when are the differences significant?

Paragraph 5.2.1 have been added to define a threshold of what a significant different is or not, compared to the natural variability of movements. All numerical values have also been added in paragraphs 5.2.2 and 5.2.3 allowing a direct comparison betwene values and confirming the difference obtained respectively to the natural variability.

7. Could you point out the advantages and limitations of the methods used, and indicate when to choose each method?
This type of approach will increase the usefulness and citation of the manuscript
A paragraph have been added at the end of section 5.3. However, we suggest to always use both metrics together to get a global overview of inter-joint coordination. Especially since sometimes, the phenomenon to be observed have an effect only on one of those characteristics but not always the one we think !

REVIEWER 2 :
a limitation of the paper is that it is difficult to understand what a particular value of a score means, e.g. is 8272 a large value for JsvCRP? Also, it is not clear how to use this technique in situations with large numbers of degrees of freedom (as is the case in more naturalistic movements) where many decision need to be made (e.g. how many PCs to use)
Reguarding the first point about the difficulty to evaluate what a specific value of a score means, paragraphe 5.2.1 have been added in order to give a first idea of what is the natural variability of the metrics in this experimental protocol. This facilitates the interpretation of a JsvCRP of 8272 since it is 8 time bigger than the upper bound of the natural variability interval.
The question of the large number of freedom is addressed in the paragraphs 6.1.1 (how ùany CRP curves to look at ?) and 6.1.2 (how many PC to consider) of the discussion.

Abstract - fix "relatinshipTs" (T instead of ')
This typing mistake was corrected.

page 2 - delete "the" in the Principal Component Analysis, and "the" continuous relative phase
This was corrected in the revised paper.

page 3 - For example, [38] presented a metric - could you give some more details about what this metric is based on?
The metric presented in [38] have been detailed. 2 points have been added : what is used to compute the metric and how to interpret the result.

- [5] have introduced function PCA - they did not introduce it, it has been used in many previous works, e.g. the book of Ramsay and Silverman in 1997
"considers one joint at a time" - this is not necessarily the case, see the PCA examples in this book (and their 2009 book on Functional data analysis with R and MATLAB)
The citation have been corrected. The explanation about the sentence “one joint at a time” have been specified too in the corresponding paragraph.

CRP - "if the result is constant, the joint phases evolve in unison" - it is not obvious (to me) why this is the case, it would be helpful to explain why this is the case (or maybe provide a graph as an example)
Two figures have been added (figure 1 and 2) to illustrate cases where joints are synchronized or not.

page 4- "there is at least as many joints" → "there are"
This was corrected in the revised paper.

page 5 - "k and l repetitions" - are you referring to k and l samples taken simultaneously (i.e. as a function of time), or "k and l" repetitions of the task (and if so, which time point are you looking at)?
The meaning of k and l have been precised in section 3.1.1 as well as the timing considerations reguarding k and l.

"removing the differences in the starting position" → this is a limitation of the technique - the movements may be highly influenced by differences in starting position, this should be discussed later
The removing of the differences in starting position is valid for small offsets in the starting position. Obviously, if the starting position of the 2 datasets are completely different, it will result in a change in the task (different joint will be involved), resulting in a change of inter-joint coordination.
In paragraphe 6.1.2 of disscussion, details about de consideration of the starting position have been added.

"with respect to the coordination strategy objectified from the intial dataset" - does this mean that is the process is repeated but switching A and B different results are obtained?
Yes, since the initial PC might be oriented differently, the projection of the data in this PC will be different and so the last PCA might be completely different. This has been clarified in section 3.1.1 as well as in section 6.1.2

page 7 - "on the opposite" → should be something like "in contrast"
This was corrected in the revised paper.

- can you explain what is the significance of the sign and magnitude of the JcvPCA?
This has been clarified in section 3.1.4

page 8 - "careful consideration" - are their some guidelines for this? how can this be avoided? filtering the data first?
In paragraph 4.1.1, details have been added reguarding the considerations.

- "if the movement times are similar" - the reason for using DTW or similar is not just because the movement times may be dissimilar, but rather because the relative amount of time for parts of the movement may be different and so should be normalized first
Corrected, thank you for the precision

- such as DTW - give a reference to DTW. It would be good to note here also the use of "registration" (as is used in the functonal data analysis book) to solve this problem
A reference to the registration process has been added as well as a reference to DTW in paragraphe 4.1.1.

"are plotted together" - isn't this Figure 2C rather than 2A?
This was corrected in the revised paper.

"at each time step" (Fig 2B) - isn't this Figure 2D?
This was corrected in the revised paper.

page 9 - JsvCRP A,B - isn't this definition problematic, because if for some of the time CRP B is bigger than A, and some of the time A is bigger than B (as in Figure 2E), won't the + and - cancel out? Should it be the absolute difference instead?

Yes that was a mistake, thank you. I’ve corrected it.

- also why 100 here? shouldn't this be more general (I assume this is for 100 time points?)
Indeed, 100 was for the 100% of the movement, but making the formula more general is a good point. It has been corrected so the intregral goes form 0 to t_{mvmt} which is the last time point of the movement.

- Can you explain what is the meaning of 3.06 as the JsvCRP - is this a big or small number?
The result of JsvCRP alone cannot be analyzed. This result has to be comparend to a baseline. The explanation of the baseline calculation is provided in section 5.2.1

page 10 - what is the end effector here? did the wrist / fingers play a role?
It has been added in section 5.1.1 that the wrist is blocked using a preformed orthesis and that the model considers the center of the palm so the fingers do not plays a role in this experiment.

Figure 4 - should joint position be joint angle (if it is in radians?)
This was corrected in the revised paper.

page 11 - it would be helpful to include again here the intermediate steps in calculating jcvPCA (in the figure)
If the intermediate steps for calculating JcvPCA were included in this figure, it would become extremely large and difficult to read. We would rather leave the figure as is.

page 12 - it would be helpful to understand whether 2411 and 8272 are considered small or large numbers
Paragraph 5.2.1 have been added to give exemple of results that could be obtained just considering natural variability of movement. In this case JsvCRP natural variability in the Physiological condition is 622.3 +/- 418.6 units. So 2411 is 2 times higher than the higher bound on the natural variability whereas 8272 is 8 times higher than natural variability, indicating a drastic change in coordination strategy.

page 16 - "for example, highly skilled players" why is it better to use this measure rather than just look at the "raw" joint angles or positions?
More details have been added on paragraph 6.1.4 to enhance the importance of considering inter-joint coordination and not just raw angles or position when evaluating motor skills.

ergonomics - what sort of changes in these two numbers would be considered "good" or "bad" ?
For ergonomis, we can considere that we expect no changes between before and after the use of a new equipment, meaning that it has not disrupted the actual pattern of inter-joint coordination. This has been specified in the corresponding paragraph of section 6.1.4.

rehab - again, what sort of changes do we expect to see?
The changes expected in JcvPCA were already explained in the corresponding paragraphe of section 6.1.4. The effect expected on JsvCRP have been added.

References
reference 1 - missing article number (this journal does not use page numbers)
reference 7 - missing article number (this journal does not use page numbers)
reference 8 - missing a journal
reference 13 - missing details
reference 20 - journal name appears twice

reference 23 - missing article number (this journal does not use page numbers)
reference 25 - missing article number (this journal does not use page numbers)
reference 28 - missing article number (this journal does not use page numbers)
reference 32 - missing details
reference 33 - missing article number (this journal does not use page numbers)
reference 44 - missing article number
reference 50 - missing article number
reference 55 - missing article number
reference 59 - missing article number
reference 60 - missing article number
All references have been corrected in the revised article

Figure 3 - Hand's projected should be hand's projection
This was corrected in the revised paper.

We hope these corrections will meet your expectations. Once again, we appreciate the opportunity to revise and resubmit our manuscript. We are committed to addressing the reviewers' comments comprehensively and improving the quality of our work for potential publication in PLOS ONE.

Thank you for your continued consideration.

Sincerely,

Océane DUBOIS
