# Supplementary material for: JcvPCA and JsvCRP: A set of metrics to evaluate changes in joint coordination strategies
Source: PLoS One. 2025 Aug 5;20(8):e0325792. doi: 10.1371/journal.pone.0325792 (PMC12324137; doi:10.1371/journal.pone.0325792)
Supplement: Attachment — Submitted filename: ResponseToReviewer2.pdf [file pone.0325792.s006.pdf]

Dear Professor Di Marco and Reviewers,

We sincerely appreciate the time and effort you have dedicated to evaluating our manuscript, "JcvPCA and JsvCRP: A Set of Metrics to Evaluate Changes in Joint Coordination Strategies" (Manuscript ID: PONE-D-24-06791R1). We are grateful for the insightful comments, which have helped us enhance the quality and clarity of our work. Below, we provide detailed responses to each of the reviewers' comments.

Reviewer 3 : In this paper, the authors propose two indices based on PCA and CRP, respectively, to quantify joint coordination during movements. The aim of the study is interesting, and the paper does not present major technical or scientific issues. However, it is not well-structured, making it difficult to understand.

A scientific paper should include the following sections:

• Introduction
• Materials and Methods
• Results
• Discussion
• Conclusions

This paper contains numerous sections and subsections that do not strictly follow this structure. Therefore, I recommend extensive editing to improve clarity.

Starting with the Introduction section, the introduction should:

• Provide background that puts the manuscript into context and allows readers outside the field to understand the purpose and significance of the study
• Define the problem addressed and explain its importance
• Include a brief review of key literature
• Highlight any relevant controversies or disagreements in the field
• Conclude with a brief statement of the overall aim of the work and a comment on whether that aim was achieved

I suggest that the authors remove all subsections related to the introduction to ensure the key points outlined above are clear. Sections 1.2.1 and 1.2.2 could be summarized using references to papers that explain PCA and CRP.

We greatly appreciate your feedback on the structure of the manuscript. We agree that a clear and logically organized structure is crucial for accessibility and comprehension. In response, we have undertaken significant revisions to improve the organization of the manuscript and ensure it aligns with conventional scientific formatting.

Introduction:

We recognize that the introduction section required substantial refinement. In response to your comment, we have completely rewritten the Introduction to streamline its presentation and ensure clarity. Specifically, we have removed the subsections in line with your recommendation, thereby eliminating unnecessary segmentation. Instead, we provide a concise narrative that integrates background context, the problem at hand, its significance, a brief review of key literature, and our study's aim.

To improve readability and ease of understanding, we have replaced the first two figures with a single figure summarizing both the PCA and CRP metrics, simplifying these complex concepts

without overwhelming the reader with excessive text. Additionally, we have added references to key papers explaining PCA and CRP, ensuring that these important concepts are sufficiently contextualized for readers outside the field.

Additionally, I recommend removing Section 2 (Notations) from the main text and turning it into an appendix.

Regarding your suggestion to move the Notations section to the appendix, we fully agree and have implemented this change. The Notations section has now been moved to supplementary material, ensuring that the main text remains focused and streamlined. However, to preserve essential clarity, we have retained key details, such as the recommended number of principal components (PCs) to retain, which has been relocated to the beginning of Section 2.1.

Materials and Methods
It appears that Section 3 marks the beginning of the Materials and Methods section. This section requires reformatting as it is too confusing and does not allow readers to replicate the procedure proposed by the authors. I suggest structuring it as follows:
2 Materials and Methods
2.1 Joint contribution variations based on PCA reprojection (JcvPCA) and explanation of the calculation
2.2 Spatio-temporal synchronization between joints based on CRP (JsvCRP) and explanation of the calculation
2.3 Application to Experimental Data

We truly appreciate this suggestion, which we believe strengthens the clarity and usability of our work. As a result, we have thoroughly restructured the Materials and Methods section to adhere to a clearer, more coherent framework. The revised section is now organized as follows:

- **Mathematical Framework for Joint Contribution Analysis Using PCA Reprojection (JcvPCA)** (Page 3)
- **Mathematical Framework for Spatio-Temporal Joint Synchronization Using CRP (JsvCRP)** (Page 5)
- **Data collection for validation of joint coordination metrics** (Page 7): This section is now subdivided to separately describe the generation of both simulated and experimental datasets. The description of the simulated dataset, which was originally part of the Results section, has now been appropriately moved here.
- **Data processing and analysis using joint coordination metrics** (Page 8): This section describes the application of the metrics to the generated datasets.

This restructuring ensures that each step in our methodology is clearly defined, making it easier for readers to follow the approach and replicate the analysis.

Results
The results section is completely missing. The authors should separate the results from the methods section and provide appropriate commentary.

We acknowledge and regret this oversight, and we are grateful for your constructive suggestion. In response, we have now added a dedicated Results section (Section 3, Page 9) to clearly differentiate the results from the methods. The results are divided into two subsections:

1. Validation using the simulated dataset
2. Validation using the experimental dataset

This division ensures that the experimental findings are presented in a clear and coherent manner, separated from the detailed methodological explanations. An additional paragraph on the way to use both JcvPCA and JsvCRP together to analyse inter-joint coordination data has been added at the end of the Results section (3.3 Interpretation of JsvCRP and JcvPCA together to characterize coordination schemes)

Discussion

The discussion is very clear and well-written. However, I question why the section is titled Discussion and Conclusions when a separate Conclusions section follows later. Ensure consistency in this regard.

We appreciate your attention to detail and agree that consistency in section titles is important for clarity. As per your suggestion, we have modified the title of the “Discussion and Conclusions” section to simply Discussion, ensuring alignment with common academic practices. The conclusions are now appropriately placed in a separate Conclusions section at the end of the manuscript, ensuring clarity and a clean distinction between the discussion of results and the study's final remarks.

Bibliography

Pay attention to the formatting of the references. The first citation in the text appears as number 54, but it should be 1. I suggest updating the references to follow the journal's style.

We appreciate your observation regarding the formatting of the references. As recommended, we have carefully reviewed and updated the reference list, ensuring that citations are now numbered sequentially according to their first mention in the text. Additionally, we have formatted the bibliography to comply with the journal’s style guidelines.

We have added a paragraph in the Discussion section to clearly emphasize that the JcvPCA and JsvCRP metrics evaluate variations in joint coordination strategies, and we specifically clarify that JcvPCA is not designed to compare fundamentally different coordination strategies. This addition aims to ensure that readers fully understand the context and limitations of the proposed metrics.

We trust that these revisions adequately address your concerns. We are grateful for your thorough review, which has greatly contributed to improving the manuscript. Please let us know if any further modifications or clarifications are required.

Thank you once again for your time and thoughtful feedback.

Sincerely,

Océane DUBOIS
