# Supplementary material for: JcvPCA and JsvCRP: A set of metrics to evaluate changes in joint coordination strategies
Source: PLoS One. 2025 Aug 5;20(8):e0325792. doi: 10.1371/journal.pone.0325792 (PMC12324137; doi:10.1371/journal.pone.0325792)
Supplement: Attachment — Submitted filename: ResponseToReviewers3.pdf [file pone.0325792.s007.pdf]

Dear Editor,

We thank you and the reviewers for the time and effort dedicated to evaluating our manuscript. We greatly appreciate Reviewer's thoughtful and constructive feedback, and we have carefully addressed all points raised. Below, we provide a detailed response to the reviewer's comments and the editorial request regarding the references.

Editorial Request: References

We have carefully reviewed our reference list to ensure it is complete and correct. We have added all DOIs when possible. No retracted papers remain in the reference list. Accordingly, the revised reference list reflects these updates.

Reviewer #3 Comments and Author Responses

Comment 1: *"Just out of curiosity, I wanted to know what the rationale was for choosing the number of PCs as p+1 instead of, for example, the number of PCs that explain at least 95% of the variance?"*

Response:
We thank the reviewer for this insightful question. In most cases, selecting the first $p+1$ principal components captures at least 95% of the variance, aligning well with common practices. However, our rationale for this choice stems from task-specific considerations. We framed the analysis in terms of the degrees of freedom necessary to perform the task rather than relying solely on variance explanation. This approach helps avoid overfitting or including extraneous components, especially in instances where participants employ atypical strategies that may introduce noise or irrelevant variability into the data.

Comment 2: *"At the beginning of page 7, there are typos in the sentence 'Experimental protocol The participant was asked to reach each of the 3 targets 5 times using different coordination strategies.' There is a lack of punctuation, and some words are in bold."*

Response:
We appreciate the reviewer pointing this out. The formatting issues and punctuation errors in this sentence have been corrected. The revised sentence now reads:
*"**Experimental protocol :** The participant was asked to reach each of the three targets five times using different coordination strategies."*

Comment 3: *"The numbering of the figures should follow the order of appearance in the text, but figure 4 is mentioned first, then figure 3, and then figure 2. I recommend rearranging them."*

Response:
Thank you for highlighting this oversight. We have revised the manuscript to ensure that figures are introduced in numerical order. The figure references have been updated accordingly to reflect the proper order of appearance.

We hope that the changes made fully address the comments and suggestions provided. We are grateful for the opportunity to revise our manuscript and for the constructive feedback that has helped us improve the clarity and rigor of our work.

Sincerely,

Océane Dubois
